# Chitosan-Based Bionanocomposite Films Prepared by Emulsion Technique for Food Preservation

**DOI:** 10.3390/ma12030373

**Published:** 2019-01-25

**Authors:** Elena Butnaru, Elena Stoleru, Mihai Adrian Brebu, Raluca Nicoleta Darie-Nita, Alexandra Bargan, Cornelia Vasile

**Affiliations:** Physical Chemistry of Polymers Department, “Petru Poni” Institute of Macromolecular Chemistry, 41A Gr. Ghica Voda Alley, RO 700487 Iasi, Romania; elena.parparita@icmpp.ro (E.B.); bmihai@icmpp.ro (M.A.B.); darier@icmpp.ro (R.N.D.-N.); anistor@icmpp.ro (A.B.)

**Keywords:** chitosan, rosehip seed oil, montmorillonite nanoclay, antibacterial, antioxidant

## Abstract

Biopolymer nanocomposite films were prepared by casting film-forming emulsions based on chitosan/Tween 80/rosehip seed oil and dispersed montmorillonite nanoclay C30B. The effect of composition on structural, morphological characteristics and, mechanical, barrier, antimicrobial and antioxidant properties was studied. The presence of rosehip seed oil in chitosan films led to the formation of flexible films with improved mechanical, gas and water vapour barrier properties and antioxidant activity. The in vitro antibacterial tests against Escherichia coli, Salmonella typhymurium, and Bacillus cereus showed that the chitosan/rosehip seed oil/montmorillonite nanoclay composites effectively inhibited all the three microorganisms.

## 1. Introduction

The global food market has strict demands regarding food safety and quality. Nowadays, society lives in a continuously changing environment, which sometimes has an important impact on the food industry. Currently, consumers prefer foods with natural food additives because these are correlated with various health benefits. This phenomenon has increased interest in searching for natural compounds with bioactive properties to preserve the quality of different types of foods. Biopolymer films could be a viable solution for some of the problems arising from the use of traditional food plastic packaging materials (e.g., originating from fossil resources, limited ability for bioactive behavior, very low biodegradability). Biopolymers with film forming properties [1,2] are suitable materials for adding different additives, such as antioxidants [3], antifungal [4] or antimicrobial agents [5], etc. Specifically, several biopolymer materials such as cellulose, starch, alginate, chitosan or pectin have good film forming capacity. Additionally, using natural polymers as coatings can prolong the shelf life of foods by controlling water loss, oxidation, or color changes, but these natural polymers have poor water vapor barriers due to their high hydrophilic nature [6]. Many studies have been conducted on edible bio-based films to demonstrate their capacity to avoid oxygen penetration to the food material or water absorption by the food matrix, loss of flavor, and solute transports [7]. One of the most promising edible active films is the one based on chitosan because of its particular physicochemical properties. Chitosan is a modified, cationic, non-toxic carbohydrate polymer, obtained through chitin deacetylation and has a complex chemical structure formed by β-(1,4) glucosamine units as its main component (>80%) and N-acetyl glucosamine units (<20%), randomly distributed along the chain. It presents some important advantages over other matrices used as edible films packaging materials such as excellent biocompatibility and biodegradability, non-toxicity, good antimicrobial activity (against bacteria and fungi), oxygen impermeability and film forming properties [8,9,10,11]. Chitosan has been approved as a food additive in some countries from Asia, like Japan (1983), Korea (1995) or China (2007). In Europe, chitosan is still not permitted as food contact material; although the European Food Safety Authority decided that chitosan is sufficiently well characterized [12]. There is a good opportunity of using chitosan-based materials as films and coatings since new regulation dealing with active and intelligent packaging was issued by the European Food Safety Authority (EFSA) [13]. The antimicrobial activity of chitosan is determined by its cationic nature. Therefore, the positively charged amino groups (NH_3_^+^) can interact with negatively charged microbial cell membranes of microorganisms. However, the antimicrobial effect against Gram-positive or Gram-negative bacteria was found to be questionable in many studies. In some reports it was found that chitosan generally has stronger effects on Gram-positive bacteria (*Listeria monocytogenes*, *Bacillus megaterium, Bacillus cereus, Staphylococcus aureus*) than on Gram-negative bacteria (*Escherichia coli, Salmonella typhymurium,* etc.) [14,15]. Due to the higher hydrophilicity in Gram-negative bacteria, it was demonstrated that this type of bacteria were most sensitive to chitosan [16]. Even though chitosan has good film forming properties, a selective permeability to gasses (CO_2_ and O_2_) and acceptable mechanical properties, the highly permeability to water vapor limits its use in contact with food. Accordingly, different methods have been used to improve the physical properties of biopolymer based films. For example, good results in increasing the hydrophobicity have been obtained by addition of neutral lipids, fatty acids, waxes, or clay [8].

Vegetable oils have received great attention from food scientists due to their antioxidant and antimicrobial activity. Rosehip seed oil can be obtained from wild roses (*Rosa moschata* L., *Rosa rubiginosa* L. or *Rosa canina Herrm*.) from Rosaceae family, bushes, which grow in various areas around the world. Rosehip seed oil has a subtle woody scent and color that can range from golden yellow to light orange. It has been found that rosehip seed oil is an important and inexpensive source of unsaturated fatty acids, the most abundant being linoleic (54.41–48.64%), followed by linolenic (17.14–18.41%) and oleic acid (14.71–18.42%) [17]. Rosehip seed oil also contains a variety of chemical compounds (mainly carotenoids, tocopherols, squalenes, phytosterols, phenolic compounds, fatty acids) which provide its antioxidant and antimicrobial activity [18]. Rosehip seed oil is becoming popular in cosmetic and other high valuable applications such as in the pharmaceutical industry, due to its antioxidant properties [19]. However, there are very few examples of the use of rosehip seed oil in the agro-food industry [19,20] or food packaging [21]. To confer superior antibacterial and antioxidant activities, barrier and mechanical properties to chitosan films various bioactive compounds and other additives have been incorporated in chitosan matrix by emulsion/casting method [22,23,24,25,26].

Hydrophilic (D-glucosamine) and hydrophobic parts (N-acetylated residues) in chitosan molecules offer to it the ability to form emulsion systems; therefore, it is used as emulsifier in the food industry, to uniformly stabilize oil droplets [22]. The emulsifying property of chitosan can be influenced by chitosan characteristics (e.g., molecular weight, degree of deacetylation) or by the type of oil used and concentration. It was found that chitosan with lower molecular weight was adsorbed more easily on the surface of the oil droplets than chitosan with higher molecular weight [23]. Rodriguez et al. [24] have studied the emulsification of sunflower oil by chitosan solution and found that the chitosan emulsifying capacity was proportional to chitosan concentration. Li and Xia also tried to understand the effects of molecular weight and degree of deacetylation on emulsifying properties of chitosan [25]. The conclusions of their study were that chitosan showed superior emulsifying activity and emulsifying stability within the range of 60.5–86.1% degree of deacetylation and that the chitosan with low molecular weight exhibited better emulsifying activity than that with high molecular weight. In the present study, a medium molecular weight chitosan with a deacetylation degree (DD) of 83.2% was selected. The vegetable oil will also act as a plasticizer for chitosan, increasing the flexibility of the final product, which is important when applying as wrapping on a food product [26]. However, the mechanical and water vapor barrier properties of polysaccharides-based films are, in general, poor. An alternative to improve these properties is the addition of layered silicates nanoparticles (e.g., sodium montmorillonite). Several studies found improvement in mechanical [26] and barrier properties [27] of chitosan films containing 1–5 wt% nanoclay. 

Starting from these considerations, the objective of this research was to analyze the beneficial effect of the addition of rosehip oil to chitosan film properties intended to be used as food packages or edible films. With this aim, new bionanocomposites films based on chitosan, rosehip seed oil and montmorillonite nanoclay are obtained using a simple and original casting emulsion method. The bionanocomposites as films were investigated for their structural, morphological characteristics and, mechanical, barrier, antibacterial and antioxidant properties. All determined characteristics proved that these bionanocomposites are good potential materials for food industry. 

## 2. Experimental

### 2.1. Materials 

Chitosan medium molecular weight (CH) with 200–800 cP viscosity in 1% acetic acid, 75%–85% deacetylation degree and M_W_ = 190,000–300,000 g/mol, was provided and used as received from Sigma-Aldrich (Darmstadt, Germany). 

Cold-pressed rosehip seed oil (RSO), was purchased from S.C Herbavit S.R.L, Oradea, Romania. It was obtained as a cold-pressed (high-pressure) oil of rosehip seeds in small batches. The fruits were collected from wild rose bushes of the common dog-rose (*Rosa canina* L., species belonging to the Rosa genus in the Rosaceae family) growing in Romania. According to the producer’s specifications it has a high content of monounsaturated fatty acids: oleic (10–20%); essential polyunsaturated fatty acids: linoleic (35–50%) and linolenic (25–50%) and also palmitic and stearic acids. In this study, the rosehip seed oil was incorporated into chitosan film in a proportion of 0.75:1 (mL/g) RSO/chitosan. IC_50_ of RSO was determined by DPPH (2,2-diphenyl-1-picrylhydrazyl) method (as described below) measuring the percentage radical inhibition vs. different oil concentrations in the range of 0.5–3.5 mg/mL. IC_50_ represents the concentration of sample required to obtain a 50% radical inhibition. RSO shows a good antioxidant activity with an IC_50_ as low as 2.3 mg/mL.

Polyoxyethylene sorbitan monooleate (Tween 80, T80), also known as Polysorbate 80 is a nonionic surfactant and emulsifier often used in foods and cosmetics, which is a viscous and water-soluble yellow liquid. Tween 80 with Mw of 1310 g/mol and more than 58% oleic acid content, obtained from Sigma-Aldrich, was added (0.125 g Tween 80/1 g chitosan) as an emulsifying agent to stabilize the hydrophobic oil droplets into aqueous chitosan solution—the resulting sample was noted as CHM. 

MMT-MTEtOH Cloisite C30B (C30B) is an organoclay derived from montmorillonite by modification with methyl, tallow (~65% C18; ~30% C16; ~5% C14), bis-2-hydroxyethyl and quaternary ammonium and was selected to improve the mechanical properties of chitosan-based films. It was obtained from Southern Clay Products (Gonzales, TX, USA) and it has 75% clay content, a moisture content of <2%, density of 1.98 g/cm^3^, average particle size of 6 μm, basal spacing d_001_ = 18.5 Å, and modifier concentration 90 meq/100g clay. It has two functional groups (hydroxyl groups and long alkyl chains linked with quaternary ammonium groups) [28] and it has been approved by the Food and Drug Administration (FDA) for use in contact with food.

### 2.2. Emulsion Preparation

Chitosan solutions (3%, w/v) were prepared by dispersing chitosan powder in acetic acid solution (5%, v/v) under magnetic stirring, at room temperature (23 ± 2 °C) for 72 h. To obtain the chitosan/rosehip seed oil (CHM/RSO) emulsion, 2 mL of rosehip seed oil and 0.375 g Tween 80 were added to the 85 mL chitosan solution (3%, w/v). The chitosan/rosehip seed oil/C30B (CHM/RSO/C30B) system was obtained by adding 0.09 g C30B to chitosan/rosehip seed oil/T80 mixture as described above. The obtained solutions were homogenized for 2 min with an ultrasonic processor UP50H (Hielscher—Ultrasound Technology, Teltow, Germany) using a power of 50 W at 30 kHz.

### 2.3. Film Preparation by Solvent Casting Method

**Figure 1:**
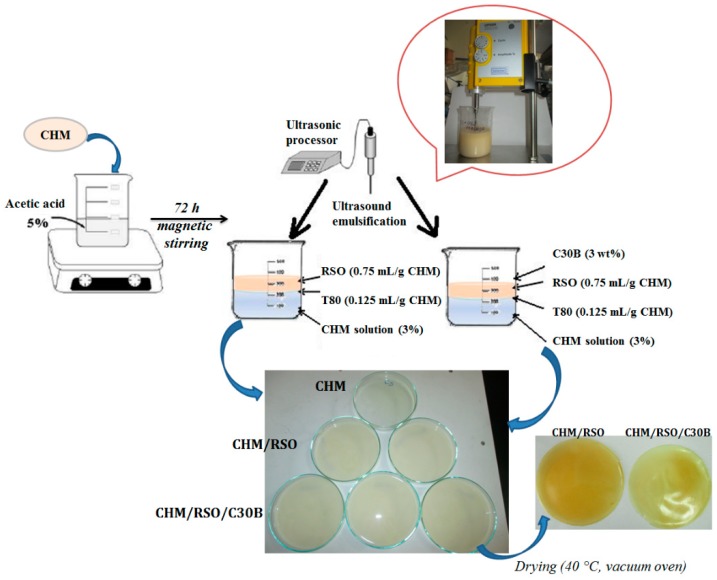
Schematic representation of the rosehip seed oil/C30B encapsulation into chitosan matrix by emulsion/solvent casting method.

The films were obtained by casting 50 mL of binary or ternary mixtures in glass Petri dishes (153 cm^2^), drying first at 25 °C in a forced-air oven for 24 h and then at 40 °C in a vacuum oven. In all cases, films with relatively uniform thickness of 0.148–0.193 mm were obtained. Prior to analyses, the films were conditioned in desiccators for 2 days at 22 °C, over a saturated solution of NaBr (58% relative humidity). The schematic representation of the encapsulation of rosehip seed oil and C30B into the chitosan matrix by the emulsion/solvent casting method is presented in Figure 1. All the films were easily removed from the cast Petri dishes and showed smooth surfaces. As a reference sample, film obtained by casting the chitosan solution containing only Tween 80 (CHM) was used. 

### 2.4. Investigation Methods

#### 2.4.1. Droplet Size Measurement and Zeta Potential Analysis

Using dynamic light scattering (DLS) technique the samples hydrodynamic radius was determined by a Zetasizer instrument (Nano ZS model, Malvern Instruments, UK), equipped with a red laser 633 nm He/Ne. The device uses technology that reduces the multiple scattering effects, which is based on a non-invasive back scatter (NIBS). The technology used is a non-invasive one, with the optics not being in contact with the sample, and backscattered light being detected. The Mie method was used during measurements over the whole recording range and comprised between 0.6 nm and 6 μm. Samples were diluted in double distilled water, each analysis consisting of three replicates and each determination was analyzed twice. The apparent hydrodynamic diameter (*D_H_*) of samples was determined using Equation (1):

(1) $$D_{H}=\frac{kT}{3\pi \eta D},$$

where *D_H_* is the hydrodynamic diameter (nm), *k*—Boltzmann constant (J·K^−1^), *T*—temperature (K), *η*—viscosity (Pa*s), *D*—the diffusion coefficient (m^2^/s). The hydrodynamic diameter is related on the intensity means, and not a mass or number mean, being calculated from the signal intensity. 

Zeta Potential (ξ) (in mV or V) was determined by using the Smoluchowski relationship:

ξ=ημ/ε, and kα>>1, where *η*—the medium viscosity (Pa·s), *μ*—electrophoretic mobility (m^2^/SV), *ε*—dielectric constant, *k* and *α*—Debye–Huckel parameter and particle radius.

#### 2.4.2. ATR-FTIR Spectroscopy

The attenuated total reflection-Fourier transform infrared spectroscopy (ATR-FTIR) technique was used to determine the samples molecular structure. Using a Bruker VERTEX 70 spectrometer the background and samples spectra were recorded in absorbance mode in the 600 to 4000 cm^−1^ wavenumber range, at a 4 cm^−1^ resolution, by performing 64 scans and the ATR module being equipped with a 45° ZnSe crystal (penetration thickness is around 100 µm). The processing of spectra was achieved using OPUS and ORIGIN programs. For each sample, the evaluations were made on the average spectrum obtained from three recordings.

#### 2.4.3. Scanning Electron Microscopy (SEM)

Scanning electron micrographs were obtained with a QUANTA 200 scanning electronic microscope (FEI Company, Hillsboro, OR, USA), at an accelerating voltage of 20 kV, which has an integrated EDX system, GENESIS XM 2i EDAX (FEI Company, Hillsboro, OR, USA) with a SUTW detector. The film surfaces were examined as such (without the metal coating), being recorded at different magnifications that are shown on the SEM images.

#### 2.4.4. Mechanical Testing

Tensile tests were carried out by means of an Instron testing machine (model 3345) with a loading cell of 5 kN, according to EN ISO 527-2/2012. The tested samples of 1 mm thickness were conditioned at 23 °C and relative humidity of 50%. A loading speed of 10 mm/min and a 40 mm gauge length were used for mechanical testing. The following tensile properties were assessed: Young’s modulus, tensile strength at the break and strain at break. For each measurement, five replicates were used and the average data are presented further.

#### 2.4.5. Gas Permeability Tests

Oxygen and carbon dioxide permeability measurements were performed on triplicate samples using a manometric gas permeation tester (Lyssy L100-5000, Systech Illinois, Johnsburg, Illinois, USA), which uses the dynamic measuring principle based on pressure change due to gas transmission through films. The experimental tests were conducted at 23 °C, after the samples were conditioned for minimum 4 h. The area of tested film was 10 cm^2^. Oxygen and carbon dioxide permeability was obtained in mL/m^2^ per day. The film separates the two compartments of the measuring chamber, the lower one being evacuated down to defined pressure while a continuous flow of gas (O_2_ or CO_2_) passes through the upper compartment. Closing the valve on the line to the vacuum pump, the pressure in the lower compartment increases due to migration through the film of the gas from the upper compartment. The instrument measures the time required for the increase of pressure within two pre-defined limits and, comparing with the values measured for a standard film, calculates the permeability of the tested film.

#### 2.4.6. Dynamic Moisture Sorption

Dynamic water vapors sorption capacity for the samples was established using a fully mechanized instrument, named IGAsorp, a gravimetric analyzer manufactured by Hiden Analytical, (Warrington, UK). This device has a very sensitive microbalance able to determine the weight change as the relative humidity is varied in the sample compartment, at a constant temperature. The measurements are totally adjusted by a special software. Initially, the samples were dried at 25 °C, in a nitrogen flow (250 mL/min) till their masses were kept constant at relative humidity (RH) <1%. After that, the RH was gradually raised from 0% to 90%, in 10% humidity periods, each one having a pre-determined equilibrium time between 40 and 60 min and the sorption equilibrium was realized for each step. Then, the RH was diminished, and sorption/desorption curves were recorded. Equilibrium moisture content (EMC) values represent the average of three different determinations. 

#### 2.4.7. Antibacterial Tests 

For testing the bactericidal activity of chitosan emulsion films, three different food pathogenic bacteria including Salmonella typhimurium ATCC-14028, Bacillus cereus ATCC-14579 and Escherichia coli ATCC-25922 were used. ATCC cultures were presented in lyophilized form and were reconstituted following the rules set out specific standards: SR EN ISO 11133/2014—Microbiology of food, animal feed and water—Preparation, production, storage and performance testing of culture; ILAC G9/2005—Guidelines on the selection and use of reference materials; SR EN ISO 7218—A1/2014—Microbiology of food and animal feeding stuffs—General requirements and guidance for microbiological examinations—Amendment 1.

The reconstituted cultures were subcultured to obtain replicate reference stock cultures and the reference stock culture was further subcultured to obtain the working stock culture. From this working stock culture bacterial suspensions of 0.5 McF (McFarland units measured with a densitometer) were obtained, which were serial diluted in order to achieve concentrations of about 10^2^–10^3^ CFU/0.1 mL. These suspensions were used for testing antibacterial activity of the surface of the chitosan films obtained by emulsion method. Sterilization of the polymeric samples was made in an autoclave at 110 °C and 0.5 bars for 20 min. After sterilization, a certain volume of bacteria suspensions was added dropwise to the surface of each 1 × 1 cm^2^ chitosan-based films placed on Petri dishes and thermostated at 37 °C. After 24 h and 48 h, using sterile tampons moistened in peptone water, the specimens from the test surfaces were collected and were seeded on the surface of specific culture media for each food pathogenic bacteria as: XLD (Xylose Lysine Desoxycholate agar) for *Salmonella typhymurium*; BACARA® (chromogenic media and method for the enumeration of *Bacillus cereus* in food products, *B. cereus* colonies turn pink-orange with an opaque halo); and VRBG (Violet Red Bile Glucose Agar) for *E. coli*. The Petri dishes with inoculated culture media were incubated at 37 °C, and after 24 h the specific colonies of each bacterial species were counted. Antibacterial inhibition was determined by the colonies counting method. Reduction in the number of CFU represented the antibacterial activity by comparison with sterile inert film used as control sample. Each experiment was realized in triplicate.

The following standardized methods of bacteriology procedures were used, according to standards in force:

- SR EN ISO 21528-2/2007, Microbiology of food and animal feeding stuffs—Horizontal methods for the detection and enumeration of Enterobacteriaceae—Part 2: Colony-count method—*E. coli*;
- SR EN ISO 7932/2005, Horizontal method for the enumeration of presumptive *B. cereus*—Colony-count technique at 30 °C;
- SR EN ISO 6579/2003/AC/2004/AC/2006, 2007, Horizontal method for detection of bacteria of the genus *Salmonella* spp;

#### 2.4.8. DPPH Radical Scavenging Assay

The radical scavenging activity (RSA) of CHM/RSO and CHM/RSO/C30B films was measured using the 2,2-diphenyl-1-picrylhydrazyl (DPPH) radical. Free radical scavenging method is the simplest, inexpensive and widely used method to measure the ability of compounds to act as free radical scavengers or hydrogen donors, and to evaluate antioxidant activity of foods [29]. The DPPH is a stable free radical, which shows a strong absorption band at 517 nm. When the stable DPPH radical accepts an electron from the antioxidant compound, the violet color of the DPPH radical is reduced to yellow colored diphenylpicrylhydrazine radical [30]. 

A total of 300 mg of each sample was used to perform the assay. This was placed in a vessel containing 10 mL of chloroform under continuous stirring for 24 h. From each mixture a 1.5 mL volume was extracted, which was mixed with 3 mL of ethanolic DPPH solution (1.5 × 10^−4^ mol/L). The control sample was obtained by mixing the same volume of chloroform with 3 mL of DPPH. The reaction mixtures were left to stand for 30 min at room temperature in the dark. Afterwards, the scavenging activity of samples was estimated by measuring the absorption of the mixture at 520 nm, using a UV-Vis spectrometer (Cary 60-UV-Vis Spectrophotometer, USA) which reflected the amount of DPPH radicals remaining in the solution.

The radical scavenging activity was calculated according to the following equation:

(2) $$\%RSA=100\times \left(\frac{A_{control}-A_{sample}}{A_{sample}}\right),$$

where *A_sample_* represents the absorbance of the sample solution in the presence of DPPH, and *A_control_* is the absorbance of the standard DPPH solution. RSA values for film samples depend on film properties.

Statistical analysis was carried out using OriginPro 8 (OriginLab Corporation, Northampton, MA, USA). All data are presented as mean value with their standard deviation indicated (mean ± SD).

## 3. Results and Discussion

### 3.1. Droplet/Particle SIZE and Emulsion Stabilization

DLS results (mean droplet diameter of the o/w nano/micro sized emulsion and Zeta potential) are compiled in Table 1. Emulsions without C30B nanoclay (CHM/RSO) had bimodal droplet size distributions. The main population of droplets (that scatter around 81% of the total scattered intensity) presented diameters of 737–1250 nm and the second population had a drop average diameter of 136 nm (around 19% of the total scattered intensity). Differently, emulsions containing C30B nanoclay had trimodal droplet size distributions. The averages diameters of the detected droplet populations were 385 nm (85%), 98 nm (7%), and 63 nm (8%), respectively. C30B nanoclay added to the continuous phase of the emulsion conferred its distinct droplet size distribution, the average size diameter of the main population of droplet decrease almost threefold. 

The zeta potential of the obtained emulsions presented positive values due to the presence of cationic polysaccharide (chitosan), as expected. By comparing the positive zeta potential with mean particle diameter at nanoscale level, it could be deduced that chitosan molecules are localized at the oil–water interface and intercalated between the nonionic surfactant (T80) molecules. Therefore, the combination of steric (by nonionic surfactant) and electrostatic (by cationic chitosan) repulsions was responsible for the stabilization of dispersed rosehip seed oil droplets in the o/w nano/micro-sized emulsions. The same behavior was noticed also for other o/w chitosan-based emulsions as mentioned by Tamilvanan et al. for castor oil-based chitosan emulsion [31]. The addition of C30B nanoclay determines and increases of the positive values of ζ-potential, indicating that nanoclay is located at the oil–water interface forming a Pickering interfacial layer [32]. Additionally, the addition of C30B nanoclay into o/w emulsion decreased the particles size. 

### 3.2. Structural Characterization of Chitosan Emulsion Films by ATR-FTIR

**Figure 2:**
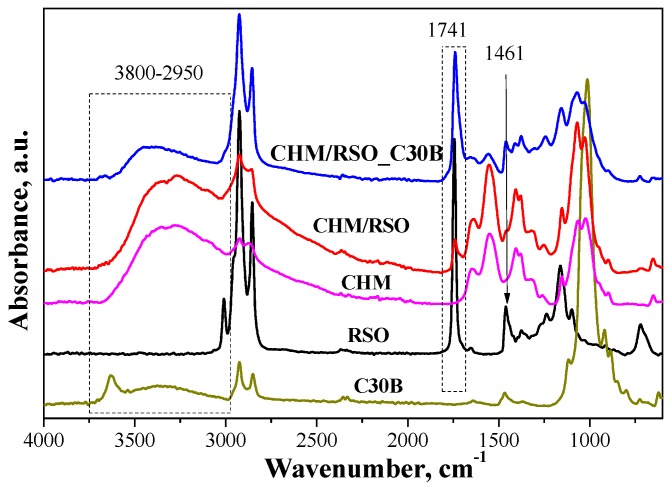
Attenuated total reflection-Fourier transform infrared spectroscopy (ATR-FTIR) spectra of rosehip seed oil (RSO), medium molecular chitosan (CHM), C30B and films obtained from emulsions.

FTIR spectrum of rosehip seed oil reveals vibrations bands at 3010 cm^−1^ assigned to =CH vibration from lipids (methyl-oleate group), and bands at 2925 and 2855 cm^−1^, which are characteristic of asymmetrical and symmetrical vibrations ν(C–H) of the CH_2_ and CH_3_ aliphatic groups from the alkyl rest of the triglycerides (found in large quantities in vegetable oils)—Figure 2. The absorption band at 1743 cm^−1^ is assigned to ν (C=O) ester carbonyl, which is a chemical group specific to oils with a high content in saturated fatty acids and short hydrocarbonated chains. The vibration band near 1654 cm^−1^ corresponds to the double C=C bond and may be correlated with the content of polyunsaturated fatty acids in the oil [33]. The band at 1461 cm^−1^ is associated the δ (C–H) deformation. Other bands are observed at 1375 cm^−1^ (deformation vibration in the phase of methylene group from lipids), 1316 cm^−1^ (δ (C–H) from resins impurities) [34], and 1237 cm^−1^ (in plane deformation vibration of =CH groups from the unconjugated cis double bonds [33]. 

The FTIR spectrum of chitosan presents a broad band between 3680 and 2980 cm^−1^, with two highlighted peaks at 3355 and 3271 cm^−1^ attributed to amine N–H stretch and O–H stretch, respectively. The band between 2980 and 2750 cm^−1^, with maximum at 2923 and 2873 cm^−1^, is assigned to aliphatic C–H stretching [35]. The band at 1647 cm^−1^ is assigned to amide C=O stretching from acetylated amino groups in chitosan. Another major absorption band with a maximum at 1549 cm^−1^ belongs to free primary amino group bending (ν NH_2_) at C_2_ position of glucosamine, which is one main group present in chitosan. The bands found in the spectrum at 1405 and 1380 cm^−1^ are attributed to the CH_2_ and CH_3_ bend. The band at 1323 cm^−1^ is due to –O–H in plane vibration of primary alcohol groups (–CH_2_–OH). The absorption bands at 1152 cm^–1^ (anti-symmetric stretching of the C–O–C bridge), 1064 and 1023 cm^−1^ (skeletal vibration involving the C–O stretching overlapped with C–N stretching vibration) are characteristics of the saccharide units of the chitosan structure [35,36]. 

FTIR spectrum of C30B clay reveals typical vibration bands responsible for stretching vibrations of the Si–O bond at 1116 cm^−1^, bending vibrations of AlAlOH at 919 cm^−1^ and AlMgOH bonds at 885–849 cm^−1^. Other absorptions bands are found at 1012 cm^−1^, assigned to stretching vibrations of Si–O–Si, and at 3631 and 3538 cm^−1^, ascribed to the free –OH stretching and the hydrogen-bonded –OH stretching, respectively. The band located at 1643 cm^−1^ for C30B organoclay is caused by the hydrogen free carbonyl, and the bands at 1467 and 1369 cm^−1^ are associated with the –CH_3_ group. Bands characteristic to –CH_2_ stretching of methylene group are found in the region located at 2924 and 2850 cm^−1^ [37].

The FTIR spectra of CHM/RSO and CHM/RSO/C30B sample reveals some characteristic bands of rosehip seeds oil incorporated into chitosan-based film, namely at 3006, 2858, 1741, and 1450 cm^−1^ that are assigned to ν (=CH), symmetrical ν (C–H) from methyl group, ν (C=O) from RC=OOR structure and to δ (C–H) deformation. Significant modifications are observed in FTIR spectra of chitosan by rosehip seed oil incorporation into emulsion-based films. The vibration absorption band given by the free O–H bond valence occurs in the region of 3590–3650 cm^−1^, and the association through polymer hydrogen bonds leads to wide bands in the region 3200–3600 cm^−1^. By rosehip seed oil addition into chitosan-based films. the band associated with free bonded O–H group of chitosan is shifted to a lower wavenumber, from 3355 to 3343 cm^−1^, indicating interaction by hydrogen bonds between chitosan and rosehip seed oil. The band ascribed to primary amino group bending (ν NH_2_) of chitosan is shifted to higher wavenumbers by RSO incorporation (from 1549 to 1554 cm^−1^). Most vibration bands of chitosan are shifted to higher wavenumbers by RSO incorporation and this may be due to the fact that the added components can inhibit the inter/intra molecular hydrogen bonding which existed in the neat chitosan film [38].

The obtained hybrid emulsion-based films exhibit characteristic bands associated with the isolated MgO–H stretching at 3690 cm^−1^, stretching of structural –OH at 3661 cm^−1^. Between 3650 and 3050 cm^−1^ a broad band is present, due to the fact that many specific bands of the components are overlapping, which can be associated with the stretching vibrations of –OH groups of interlayer water from C30B, N–H stretching and hydroxyl –OH stretching [36,39,40].

The FTIR spectroscopy has demonstrated the RSO and C30B clay successful incorporation into chitosan-based film. The formation of hydrogen bonds and van der Waals interactions that appear on the phase boundaries, indicated by the band shift in FTIR spectra, influences the mechanical properties of the materials. Addition of the C30B nano-filler to the liquid system of chitosan dissolved in acetic acid solution facilitated formation of hydrogen bonds between functional groups of the chitosan matrix, mainly with amine and hydroxyl groups, and the Si–O bonds existing within the silicate layers of the nanoclay. Moreover, both hydroxyl and amine groups of chitosan may bond with Si–O–Si groups of the silicate layer of C30B clay [37].

### 3.3. Morphological Analysis

**Figure 3:**
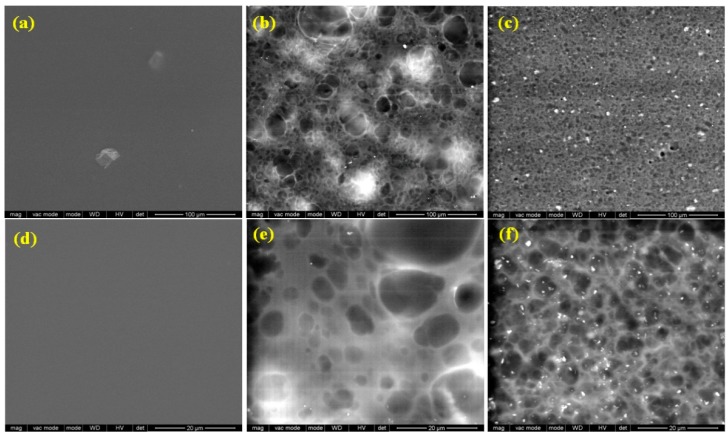
Scanning electron microscopy (SEM) images of CHM, CHM/RSO and CHM/RSO/C30B films; (**a**–**c**) scale bar: 100 µm and (**d**–**f**) scale bar: 20 µm.

Scanning electron microscopy examination reveals the morphology of the films surface obtained by the incorporation of rosehip seeds oil and nanoclay C30B into chitosan films (Figure 3). Analysis of the CHM sample reveals a smooth and continuous film structure surface (Figure 3a,d). Inspection of CHM/RSO/T80 film surface showed the presence of large spherical cavities, attributed to RSO oil droplets, that are relatively homogenously dispersed into the CHM matrix (Figure 3b,e), as revealed by SEM images. Similar surface microstructure, namely discontinuities associated with the formation of two phases, was found for quinoa protein-chitosan-sunflower oil film by Valenzuela et al. [41]. The average particle size of the RSO globules is 4.7 ± 1.6 µm, with the size of oil droplets varying from 2.1 to 7.2 µm. The good homogeneity of the films and the encapsulation of the oil are assured by the presence of the nonionic surfactant and T80 emulsifier. By C30B nanoclay addition into the emulsion system, the average particle size of the RSO droplets decrease at about 2.6 ± 0.7 µm, the oil droplets being better distributed into the emulsion-cast film (Figure 3c,f). The C30B organoclay is also well distributed into the emulsion-cast films with an average particle size of 0.44 ± 0.1 µm. 

### 3.4. Mechanical Properties

Mechanical properties are important when referring to materials proposed for applications in the packaging industry, because strength at break and flexibility ensure the integrity of films intended for food packaging. The strength at break indicates the maximum tensile stress that the film can resist while being pulled before breaking [42].

**Figure 4:**
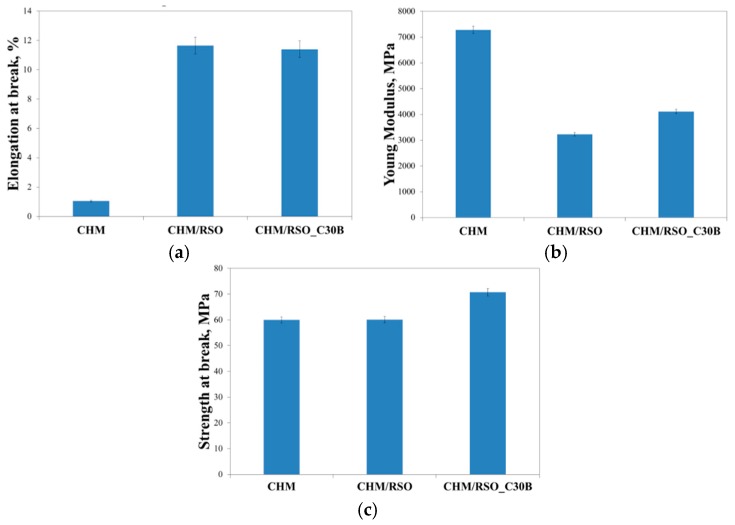
Elongation at break (**a**), Young modulus (**b**), and strength at break (**c**) of CHM, CHM/RSO and CHM/RSO/C30B emulsion-cast films.

The effect of incorporating RSO and nanoclay C30B on the mechanical properties of chitosan-based films are shown in Figure 4. 

Chitosan control film (containing T80) had the strength at break of 59.9 MPa, Young Modulus value of 7282.0 MPa and 1.05% elongation at break. Incorporation of rosehip seed oil and C30B nanoclay into chitosan films significantly increased elongation at break from 1.05% to 11.7% in case of RSO incorporation and 11.40% for RSO/C30B. The nanofiller does not change elongation at break. A high value of Young modulus was registered for the reference chitosan film, which means that it is a rigid material. On the other hand, an increase of tensile strength and a decrease of elastic modulus after the incorporation of the rosehip seed oil and C30B nanoclay can be observed in Figure 4. The strength at break values for the chitosan based-films containing RSO and C30B nanoclay, are 60.1 and 70.7 MPa, respectively and Young modulus values determined for the same samples were 3232.3 and 4110.9 MPs, respectively. This behavior could be attributed to the oil components, which act like plasticizers for the chitosan film. The role of plasticizers is to weaken the inter/intra molecular forces between polymer chains, and thereby increasing the flexibility of the films [6]. A different behavior was noticed for chitosan films formulated with clove essential oil (CEO) and halloysite nanotube (HNT), the films with CEO presented lower elongation values than the film without CEO [43]. There are also other reports indicating a reduction of the tensile parameters when a bioactive compound is incorporated in chitosan films [44,45]. The authors attributed this behavior to the presence of structural discontinuities in the chitosan matrix caused by the oil-dispersed phase. The C30B further improved the strength at break of the films. According to Krochta and De-Mulder-Johnston’s [46] film classification, values of tensile strength below 10 MPa indicate poor mechanical properties. Therefore, chitosan based-films containing RSO and C30B nanoclay show good and satisfactory mechanical properties; and the strength at break values can be considered acceptable for materials proposed for applications in bioactive food packaging.

### 3.5. O_2_ and CO_2_ Permeability

A barrier polymeric material is most commonly used in the packaging industry to stop the passage of small molecules of gases, moisture or flavors. The barrier properties of a polymeric film are important because it is necessary to preserve the quality of food and to estimate its shelf-life [47]. Generally, gas permeability depends on film microstructure (holes or discontinuities in the polymer structure), thickness, the solubility of gases in the corresponding material, and the level of arrangement of the polymer chains [42]. Oxygen and carbon dioxide are the most common permeates studied in food packaging applications. The oxygen diffusion from air into the package should be avoided because the product quality can be modified during prolonged storage due to the oxidation, which leads to changes in organoleptic properties and decreased nutritional value. The oxygen permeation through a material is calculated as the weight or volume of oxygen which is able to pass through a known area of a packaging material in a given time [48]. Like oxygen, the barrier property to carbon dioxide is also important in food packaging applications, since carbon dioxide can initiate deterioration reactions and should be removed to ensure freshness of food products.

Table 2 summarizes the results from O_2_ and CO_2_ permeability tests of the chitosan emulsion films. The values of O_2_ permeability for reference chitosan film was of 67 mL/m^2^ per day and after incorporation of rosehip seed oil and C30B nanoclay, the O_2_ permeability was higher with values of 212 and 134 mL/m^2^·per day, respectively. These kind of gas barrier features were observed also by other authors, the values of O_2_ permeability of chitosan films being comparable [41,49]. The increased values of O_2_ could be attributed to the plasticization effect of the amorphous biopolymer backbone by the water molecules [50]. The plasticizing effect tends to increase the mobility of the oxygen molecules due to the disruption of hydrogen-bonding, creating additional sites for the dissolution of oxygen [51]. The presence of C30B nanoclay leads to the improvement of CO_2_ barrier properties reaching half lower CO_2_ values for films with C30B compared to neat chitosan.

Even if the samples obtained by RSO and C30B addition into chitosan matrix presents higher oxygen permeability, the obtained values indicate that chitosan emulsion films show a superior oxygen barrier compared to a synthetic biodegradable polymer-based packaging material, polylactic acid (PLA), of similar thickness (Table 2).

### 3.6. Dynamic Moisture Sorption 

**Figure 5:**
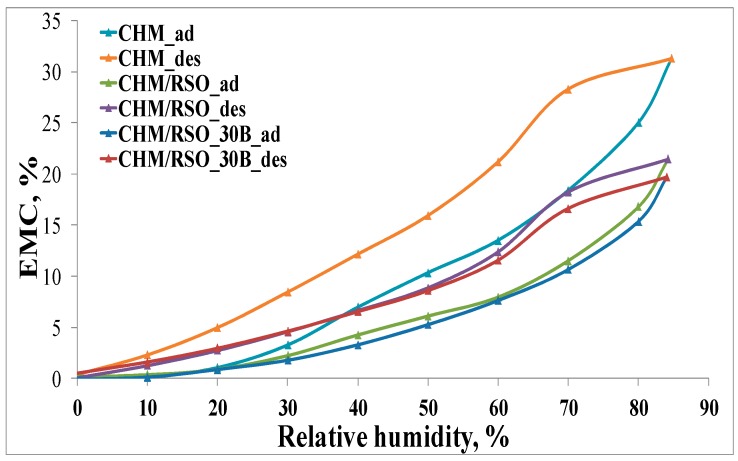
Moisture sorption isotherms (adsorption/desorption) for chitosan-based samples.

The relationship between the equilibrium moisture content (EMC) against the relative humidity (RH) at constant temperature (25 °C) for chitosan-based films is presented in Figure 5.

According to the IUPAC classification [52,53,54], the adsorption/desorption isotherms can be associated to type V curves describing adsorption on hydrophobic/low hydrophilic materials with weak sorbent–water interactions. The values of water vapor sorption capacities for all the samples are presented in the Table 3. The isotherms show hysteresis between sorption and desorption. In Figure 5, it can be seen that the final mass is the same as the initial one. The chitosan-based films showed an improvement of the water vapor barrier property by incorporation of rosehip seed oil and C30B nanoclay in the film matrix (Table 3). The EMC of rosehip seed oil–chitosan films is significantly lower than that corresponding to the reference chitosan film. This may be due to the diminished availability of amino groups of chitosan, determined by the electrostatic neutralization with the carboxylate groups of rosehip seed oil that contributes to the compactness of the film network and may cause limited access of water molecules to the hydrophilic sites of chitosan. Pereda et al. [6] observed the same behavior of EMC for olive oil–chitosan films.

Addition of C30B nanoclay into the chitosan-RSO emulsion film leads to a further slight decrease in equilibrium moisture content. This result indicates that nanoclay affects the water vapor barrier property of biopolymer films, which may be due to the fulfilling of interspaces in the chitosan matrix by nanoclay particles [55] or due to the hydrophobic nature of the C30B nanoclay [40]. 

### 3.7. Antibacterial Inhibition

**Figure 6:**
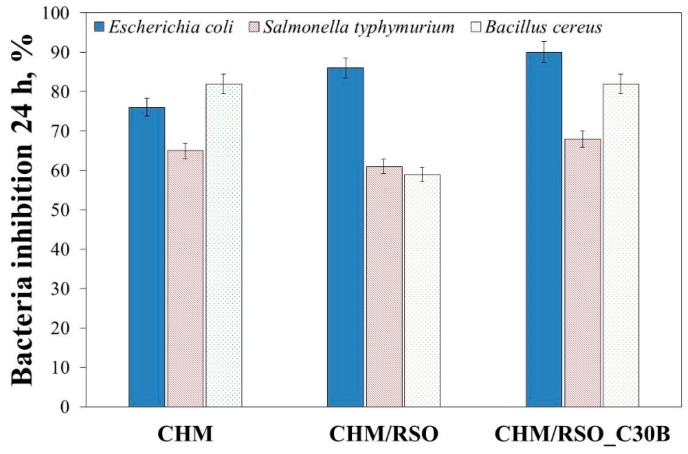
The antibacterial activity of chitosan-based bionanocomposites against *Escherichia coli*, *Salmonella typhimurium* and *Bacillus cereus*.

Two Gram-negative bacteria, namely *E. coli* and *S. typhimurium*, and one Gram-positive bacterium, *B. cereus* were used as test bacteria to examine the bactericidal properties of chitosan film and the effect of RSO, as well as C30B nanoclay addition on the antibacterial action of chitosan. These bacteria were selected because they are the common foodborne pathogens, which are responsible for most of the foodborne diseases [56]. The results of antibacterial tests are shown in Figure 6.

From the results shown in Figure 6, it can be observed that chitosan film is an effective antimicrobial agent against the tested bacteria. The results showed bacteria inhibition at 24 h, with 65% reduction for *S. typhimurium*, 73% reduction for *E. coli,* and 82% reduction for *B. cereus.* Addition of rosehip seed oil resulted in the decrease of the antibacterial action against *S. typhimurium* and *B. cereus.* On the other hand, all tested chitosan emulsion films showed pronounced antibacterial activity against *E. coli*. Addition of rosehip seed oil and C30B nanoclay resulted in the increase of antibacterial activity of the chitosan film against *E. coli*. These results are in accordance with those reported by Kamarasamy et al. [57], where a weak antibacterial effect of rosehip seed oil was demonstrated, and only against *E. coli*. Additionally, is important to mention that addition of C30B nanoclay resulted in an increase of antibacterial activity of the chitosan emulsion films against all tested bacteria (Figure 6). Similar results regarding the antibacterial property of C30B nanoclay were also reported by various authors [40,58,59] The results shown in Figure 6 indicates a synergism between chitosan, rosehip seed oil and C30B nanoclay, and that by mixing them a material with strong antibacterial property resulted. 

### 3.8. Antioxidant Activity 

**Figure 7:**
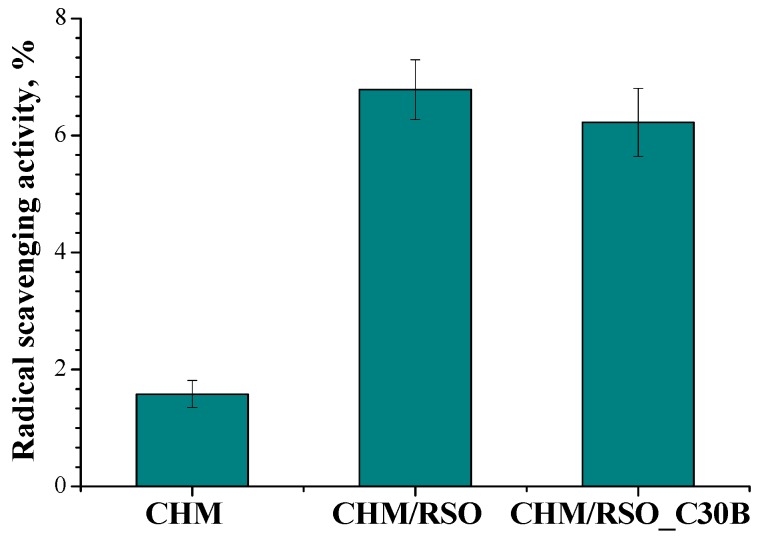
Radical scavenging activity (RSA) of chitosan-based films obtained by emulsion cast technique, determined after 30 min.

Antioxidant properties, especially radical scavenging activity, are important because free radicals have a negative effect both on foods and the human body, leading to oxidative stress, which is involved in the development of many human diseases [60,61]. As demonstrated by other authors, chitosan itself holds some antioxidant activity, mainly attributed to the capacity of residual free amino groups of chitosan to react with free radicals, forming stable macromolecular radicals and ammonium groups, but its activity is negligible compared to other bioactive compounds [49,62,63,64,65]. When rosehip seed oil was added to the chitosan film, the radical scavenging activity (RSA, %) increased up to three-fold (determined after 30 min). The addition of C30B nanoclay into the chitosan/rosehip seed oil films did not significantly affect the antioxidant activity of the samples (Figure 7). 

## 4. Conclusions

Films based on chitosan, rosehip seed oil and C30B montmorillonite were successfully prepared and characterized by the encapsulation emulsion method. The addition of rosehip seed oil, which acts also as antioxidant, antibacterial and plasticizer, was found also responsible for improving the flexibility of chitosan-based films, whereas addition of the C30B nanoclay improved the strength at break of the biocomposites. The inter/intra molecular hydrogen bonding between components, confirmed by FTIR analysis, assures the excellent integrity of the films and, as a consequence, superior gas (oxygen and carbon dioxide) and water vapor barrier properties. The results of the present study show that the chitosan emulsion films exhibited potent antibacterial and antioxidant activity, and can be considered acceptable for materials proposed for applications in bioactive food packaging. 

## Figures and Tables

**Table 1:** Zeta potential (ζ) and average diameters (d) of the emulsions’ droplets.

Sample	ζ-Potential (mV)	Droplet Sizes	
		Type of Distribution	d (nm)
CHM/RSO	24.35 ± 1.17	Bimodal	993.5 ± 256 136.1 ± 35
CHM/RSO_C30B	30.7 ± 1.31	Trimodal	385.7 ± 84 98.17 ± 16 63.11 ± 11

ζ—Zeta potential; d—average diameter.

**Table 2:** Permeability to O_2_ and CO_2_ of chitosan emulsion-cast films.

Sample	Thickness (mm)	CO_2_ (mL/m^2^ per day)	O_2_ (mL/m^2^ per day)
PLA	0.151 ± 0.012	873 ± 26.1	1308 ± 39.4
CHM	0.148 ± 0.008	45 ± 1.8	67 ± 3.35
CHM/RSO	0.237 ± 0.019	37 ± 1.1	212 ± 10.6
CHM/RSO_C30B	0.193 ± 26.19	18 ± 0.9	134 ± 6.7

**Table 3:** Equilibrium moisture content (EMC) assessed at 90% relative humidity (RH) of chitosan-based films containing RSO and C30B.

Sample	EMC (%)
CHM	31.29 ± 1.1
CHM/RSO	21.44 ± 0.8
CHM/RSO/C30B	19.70 ± 1.2

EMC—equilibrium moisture content.
